# Case report and successful management of canine aortic endocarditis caused by *Actinomyces neuii* subsp. *anitratus* (*Winkia neuii* subsp. *anitrata*)

**DOI:** 10.1186/s12917-022-03161-3

**Published:** 2022-02-03

**Authors:** G. Giannoulopoulos, K. Errington

**Affiliations:** Wilson Vet Group, Bishop Auckland, Co-Durham, DL14 7AD UK

**Keywords:** *Actinomyces*, Dog, Endocarditis, Aortic valve, *Winkia*

## Abstract

**Background:**

Canine aortic valve endocarditis carries a poor prognosis. In the current literature there are only two reports of infectious endocarditis associated with *Actinomyces*; *Actinomyces turicensis* and an *Actinomyces*-like organism. Endocarditis due to *Actinomyces neuii subsp. anitratus* (now known as *Winkia neuii* subsp. *anitrata)* has rarely been reported in humans, and to the best of our knowledge, has never been reported in dogs.

**Case presentation:**

A 4 year-3 months old female neutered Great Dane presented with lethargy, hyporexia, ‘praying position’ stance, acute onset of cherry eye and pyrexia. A subtle diastolic heart murmur was detected on thoracic auscultation and echocardiology revealed an irregular lesion adhered to the ventricular aspect of the aortic valve, suggestive of aortic valve endocarditis. Peripheral blood was collected for blood culture. Following 10 days of incubation, blood cultures yielded a growth of aerobic gram-positive filamentous rods which were further biochemically (BioMerieux API Coryne profiling strip) identified as *Actinomyces neuii subsp. anitratus*. The patient was treated with marbofloxacin and amoxicillin/clavulanic acid for five consecutive months. On repeat echogram, following treatment completion, there was no evidence of aortic valve endocarditis. To the best of our knowledge this is the first case report documenting successful treatment of aortic valve endocarditis caused by *Actinomyces neuii subsp. anitratus* in a dog.

**Conclusions:**

Despite the poor prognosis of canine infectious aortic valve endocarditis, patients with *Actinomyces neuii subsp. anitratus* infection might have a favourable outcome. It is therefore important identifying the underling infectious cause, as it may have a significant impact on prognosis and treatment outcome when it is caused by *Actinomyces neuii subsp. anitratus.*

## Background

Infective endocarditis is a life-threatening infection of the endocardium or the heart valves (valvular endocarditis) caused by microbial invasion of heart endothelium [7]. The most common bacterial causative agents include *Staphylococcus spp, Streptococcus spp., Escherichia coli, Pseudomonas aeruginosa, Bartonella spp., Corynebacterium spp.,* and *Erysipelothrix rhusiopathie* [7] [6]. Mitral valve endocarditis in a dog, due to *Actinomyces* [5] has also been reported. Canine aortic endocarditis carries a poorer prognosis with a mean survival time as low as 3 days, when the equivalent for dogs with mitral valve infective endocarditis is 476 days [8]. In humans, aortic endocarditis due to *Actinomyces spp* is rare but carries a favourable prognosis [12]. *Actinomyces neuii* has not previously been reported as a causative agent of infective endocarditis in dogs. Furthermore, successful treatment of canine aortic endocarditis could be considered rare, since the overall prognosis for these cases is grave [7].

## Case presentation

A 4 year-3 months old female neutered Great Dane presented with vague symptoms that included whining, tachypnoea and hyporexia. The patient, who was up to date with endo- and ecto-parasitic preventatives but not up to date with her vaccinations, had a history of mild chronic recurrent otitis. On presentation to the referring vets (RV) she was very nervous, had a rectal temperature of 39.9 C and mild discomfort on neck flexion sideways but not on dorso-or-ventroflexion; no other abnormalities were detected. She received a treatment trial with amoxicillin/clavulanic acid (15 mg/kg every 12 h, for 10 days), gabapentin and carprofen but remained pyrexic. She subsequently became hyporexic and displayed a ‘praying position’ stance. Presence of haemoglobin in the patient’s urine (dipstick) led to a provisional diagnosis of urinary tract infection leading to extension of amoxicillin/clavulanic acid (10 mg/kg every 12 h, for a further 4 days).

The patient presented as an emergency 4 weeks following initial presentation to the RV, with ongoing lethargy, hyporexia, ‘praying position’ stance and acute onset of third eyelid gland prolapse (also known as ‘cherry eye’) of her right eye. On examination, the patient’s heart rate was 148 beats per minute (bmp), respiratory rate was 30 breaths per minute (br.pm) and rectal temperature was 39.7°C. After careful examination and palpation no spinal, neck or joint pain was detected but she appeared sensitive when attempting to wide-open her mouth. Abdominal palpation was unrewarding. On thoracic auscultation, a previously undetected subtle grade II/VI diastolic heart murmur was noted at the left base of the heart; the rest of the thoracic auscultation was unremarkable. Mucous membranes were pink but tacky and the capillary refill time (CRT) was 2 s. Haematology revealed a haematocrit of 54%, increased white blood cell count 28.11 X 10^9^/L (neutrophilia, left shift, normal range 6.0—17 X 10^9^/L) and moderate thrombocytopenia 71 X 10^9^/L (normal range 165 – 500 X 10^9^/L). Serum chemistry and electrolytes were unremarkable and prothrombin and partial thromboplastin times were within normal range. A fresh free-catch urine sample presented by the owner was submitted for culture; in house analysis revealed urine specific gravity of 1.060; urine dipstick revealed protein (2 +), ketones (1 +), blood (1 +), leukocytes (1 +) and pH of 7.5.

**Fig. 1:**
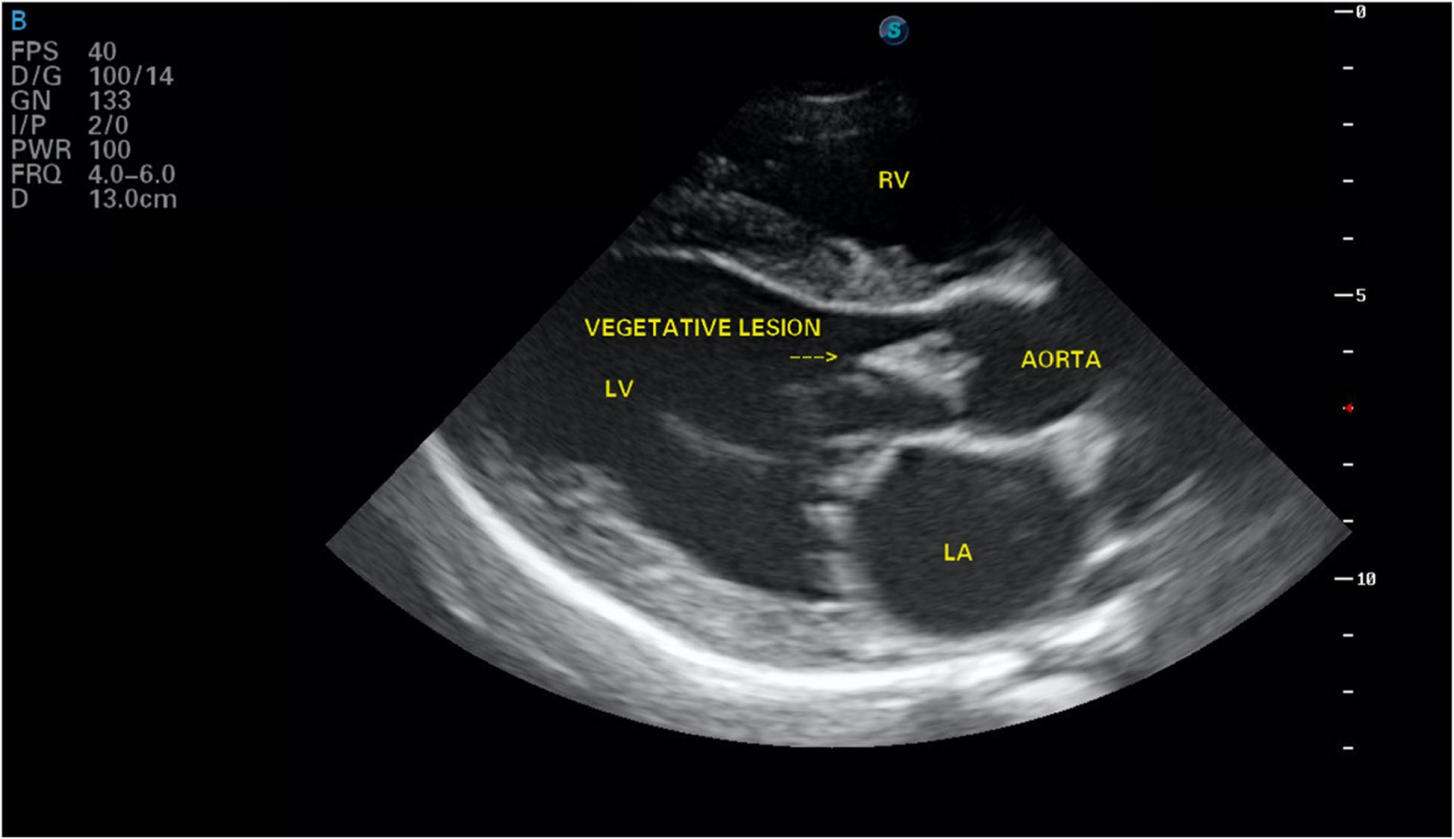
Aortic valve vegetation as seen on right parasternal long axis echocardiography (LA: left atrium, LV: left ventricle, RV: left ventricle)

**Fig. 2:**
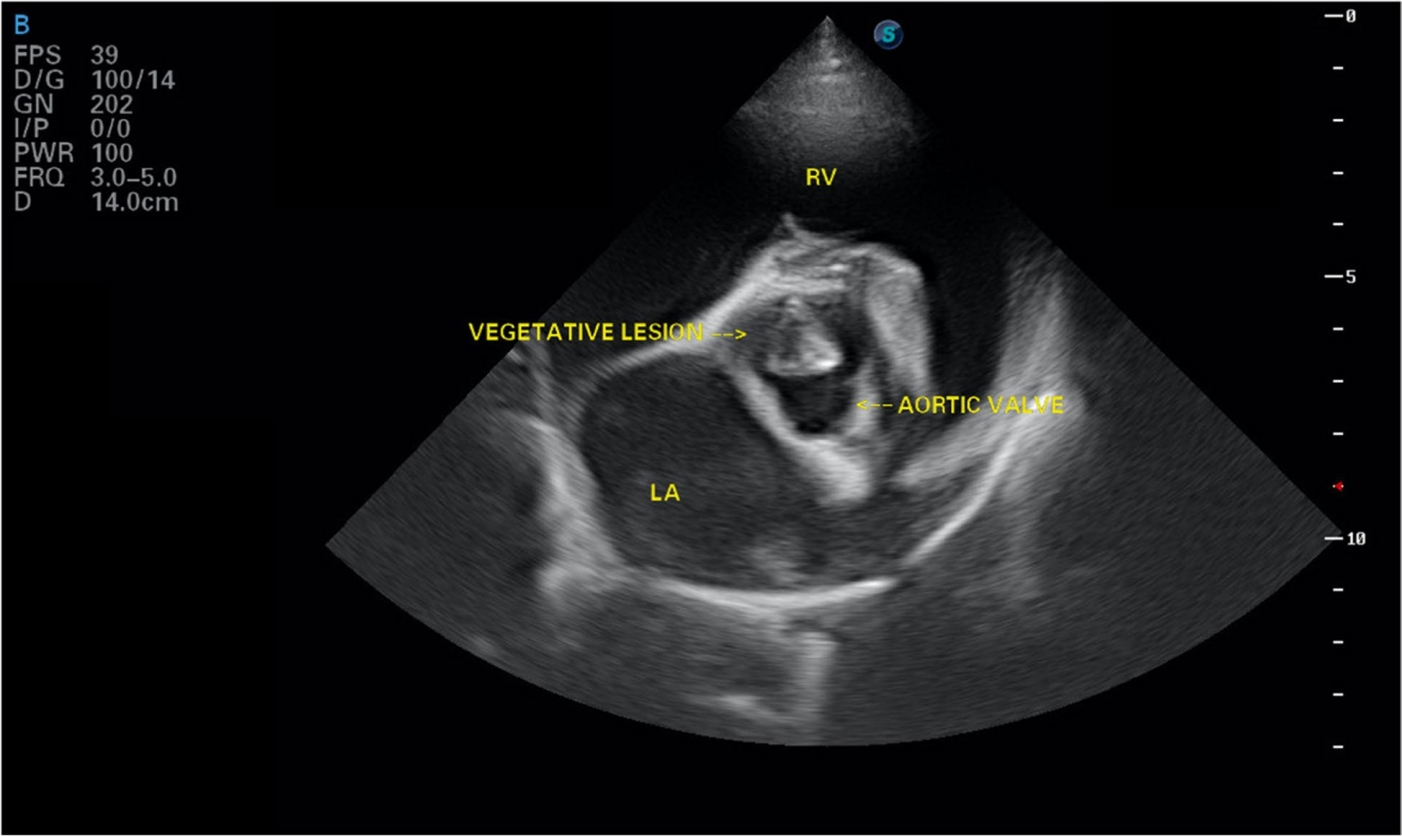
Aortic valve vegetation on short axis echocardiographic view (LA: left atrium, RV: left ventricle)

Echocardiology in the right parasternal long axis view revealed a hyperechoic, irregular triangular shaped lesion adhered to the ventricular aspect of the aortic valve measuring 22 mm in length (Fig. 1). Colour flow Doppler identified aortic insufficiency, with a turbulent jet of aortic regurgitation. Continuous wave Doppler revealed a mildly increased left ventricular outflow velocity (2.94 m/s, normal velocity < 2 m/s). There was no evidence of a subvalvular ridge. The right parasternal short axis view at the level of the aortic root showed the hyperechoic lesion in the region of the left coronary cusp (Fig. 2). The left ventricular diastolic diameter was slightly increased [(LVDd 5.7 cm, 1.83 normalised, range 1.35–1.73: LVDs 2.7 cm, 0.8 normalised, range 0.79–1.14) [2] with increased fractional shortening (FS) of 50% (normal range 28 – 45%). The left atrium to aortic diameter ratio was slightly increased (ratio of 1 to 1.8, normal < 1.6). A provisional diagnosis of aortic valve endocarditis was made. A single 5 ml blood sample was aseptically collected from the left jugular vein, placed in blood culture medium and submitted for culture. A peripheral EDTA blood sample was submitted for Bartonella qPCR. Pending results, the patient was discharged on oral amoxicillin/clavulanic acid 15 mg/kg every 12 h, marbofloxacin 2 mg/kg once daily, paracetamol 10 mg/kg every 8 h and eye drops containing polymyxin B, neomycin and dexamethasone for her cherry eye. Urine culture from the free catch sample yielded a coliform growth which was resistant to amoxicillin/clavulanic acid but sensitive to marbofloxacin. Bartonella PCR was negative. Following 10 days of incubation, blood cultures yielded moderate growth of aerobic Gram-positive filamentous rods. This gram-positive organism was further biochemically (using BioMerieux API Coryne profiling strip) identified as *Actinomyces neuii subsp. anitratus*.

The patient returned two weeks following discharge for further investigation; she was found to be normothermic, she had stopped displaying the praying position stance and her heart murmur had changed to a systolic, grade IV/VI at the base of the heart. Radiographs of her spine, head and chest were obtained and an abdominal ultrasound was carried out. A fresh urine sample was obtained via cystocentesis and submitted for culture. Arthrocentesis from multiple peripheral joints (right and left carpi and stifles, left tarsus) were performed for cytological examination and culture. The most significant findings from the radiographic study included cervical articular process joint osteoarthritis, possible C5/C6 intervertebral disc space narrowing and multiple sites of mild thoracic and lumbar spondylosis deformans. No radiographic evidence of dental, auricular, pulmonary disease or diskospondylitis were present. The abdominal ultrasonographic study was unremarkable. No evidence of inflammatory arthropathy was noted on cytology and both the urine collected via cystocentesis and joint cultures yielded no growths.

## Follow up

**Fig. 3:**
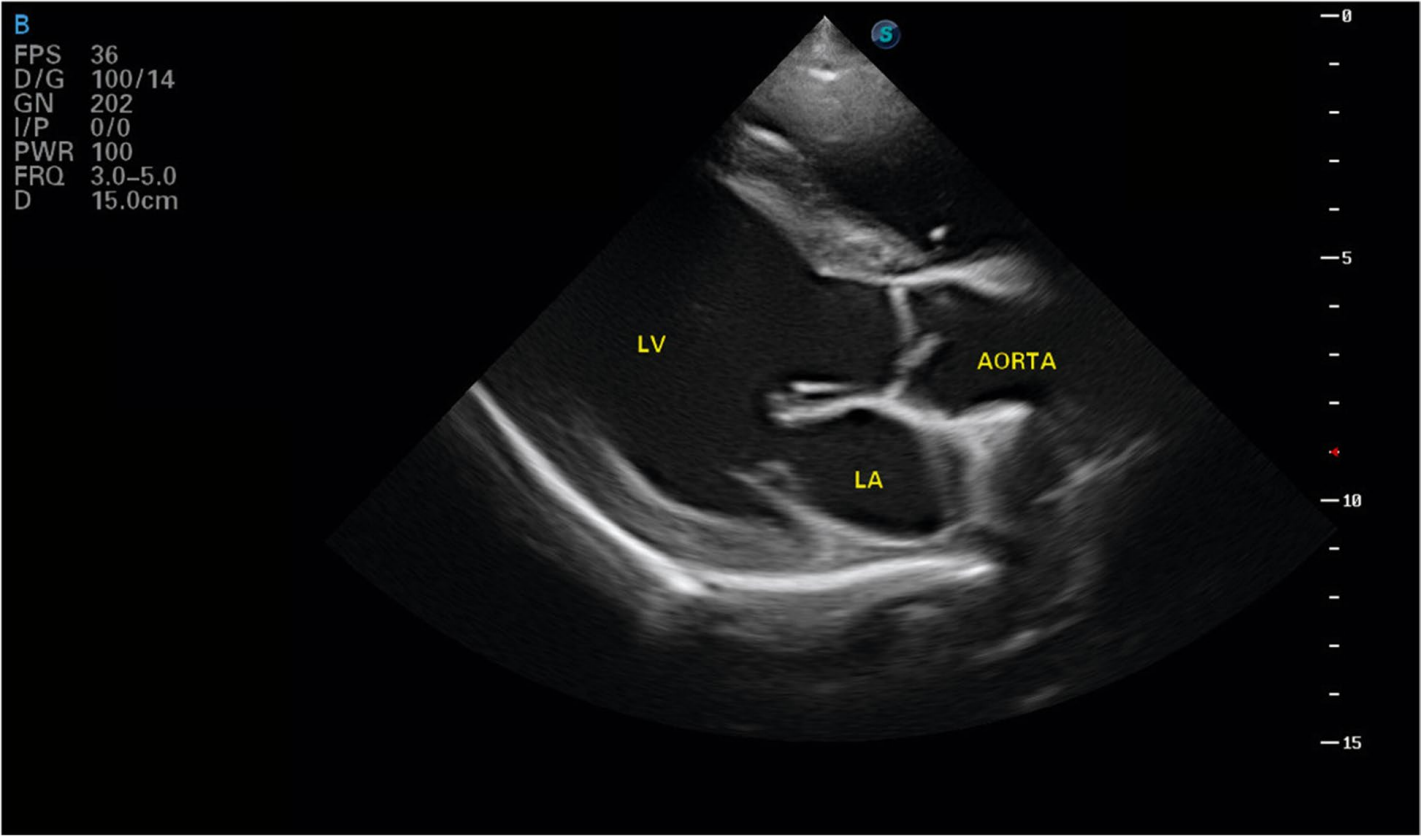
Echocardiography, following resolution of the vegetative aortic lesion (LA: left atrium, LV: left ventricle)

Five weeks after starting the combination of antibiotics the cardiac ultrasound was repeated and no changes were seen to the vegetative lesion, however there was an increase in the left ventricular size with both systolic and diastolic diameters increasing (LVDd 7.05 cm normalised 2.06 and LVDs 3.9 cm normalised 1.14). Fractional shortening had reduced and was now within the normal range (FS 42%). A further echocardiogram was performed 4 months later; the hyperechoic lesion previously adhered to the aortic valve was no longer visible (Fig. 3). In the right parasternal short axis view, the left coronary cusp of the aortic valve was seen to be irregular in shape with a vena contracta apparent during valve closure. Eccentric hypertrophy of the left ventricle had progressed with further increase in systolic and diastolic left ventricular diameter (LVDd 7.4 cm, 2.38 normalised: LVDs 5.2 cm, 1.54 normalised) and FS had reduced further to 30%.

The patient was treated with the same combination of antibiotics for a total of 5 months at which stage she no longer had echographic evidence of endocarditis and white blood cell count and serum C-reactive protein were within normal range. Both blood and urine cultures were repeated a month after the end of her treatment and both yielded no growth.

In her one year post-diagnosis follow up, the patient was doing remarkably well. Her owner reported no concerns. On clinical examination the systolic heart base murmur remained unchanged (grade IV/VI). Peripheral pulse was good quality with no pulse deficit and rectal temperature was normal. No abdominal, spinal or oral pain detected. Blood pressure was within normal limits (average 129/91 mmHg). A repeat echogram confirmed the aortic valve was still clear of hyperechoic vegetative lesions although the distortion of the left coronary cusp remained. Aortic insufficiency and raised aortic velocity (3.2 m/s) were also still present. Left ventricular diameter had reduced (LVDd 6.88 cm normalised 2.15, LVDs 4.4 cm normalised 1.26) when compared to last assessment, and FS had increased (35%).

## Discussion and Conclusions

In humans, *Actinomyces ne*uii has been isolated and cultured from abscesses, infected atheroma, infected ulcers and wounds, endophthalmitis, cases of infective endocarditis, urinary tract infections, cellulitis, infectious arthritis and from bacteriaemia [11]. Following culture, identification of the pathogen can be performed with various methodologies including 16S rRNA gene sequency and matrix-assisted laser desorption ionization-time of flight mass spectrometry (MALDI-TOF MS). Even though MALDI-TOF MS is considered a more reliable and accurate method, API Coryne test strips have been routinely used in labs for *Actinomyces spp.* identification with good accuracy [3, 10, 13]. There isn’t enough information regarding the presence of *Actinomyces neuii* in the environment but it has been found to colonize the oral cavity in humans [11]. It is unclear in our case, how the patient acquired the microorganism.

Canine infectious endocarditis is challenging to diagnose, and difficult to treat and therefore often carries a poor prognosis. In small animals mitral and aortic valves are the most commonly affected, with aortic valve endocarditis carrying a grave prognosis [7]. ‘*Actinomyces canis-like’* endocarditis [1] and *Actinomyces turicensis* endocarditis [5] have also been previously reported. To the best of our knowledge this is the first case report and successful treatment of *Actinomyces neuii subsp. anitratus*, now known as *Winkia neuii subsp. anitrata *[9], endocarditis in a dog. This patient presented with chronic pyrexia of unknown origin and with a newly diagnosed diastolic left sided heart murmur on initial referral consultation. Imaging of her spine and joint taps revealed no abnormalities associated with an underlying infectious process. Diskospondylitis can be a predisposing factor for bacteraemia, and immune-mediated disease (e.g. immune-mediated polyarthritis) can develop due to increased antibody circulation against the causative agent of the infection [7]. Urine culture from a free catch sample yielded an unspecified coliform growth of 10^3^ colony forming units (CFU) per ml. Echogram revealed a vegetative lesion on her aortic valve and blood culture yielded a growth of *Actinomyces neuii subsp. anitratus*. Despite attempting obtaining sensitivities to various antibiotics, this was not possible due to the very slow growing nature of the organism. Overall, the above aforementioned findings, fulfilled the criteria previously established for the diagnosis of canine bacterial endocarditis [7]. The patient was treated empirically with a combination of a penicillin and a fluoroquinolone [5] and following the blood culture results she remained on the same antibiotics due to clinical improvement. Even though aortic valve endocarditis carries grave prognosis [7], this patient recovered and hadn’t developed heart failure up to the time of writing of this report, 25 months following initial diagnosis. In humans, *Actinomyces* spp endocarditis is rare and these infections usually have an indolent course and favourable prognosis [12] similar to our case. C-reactive protein has previously been utilised in humans for monitoring the outcome in patients with infectious endocarditis [4]. It is not clear as to why this patient had not previously responded to amoxicillin/clavulanic acid as traditionally *Actinomyces spp* are susceptible to beta lactam antibiotics [12]. She previously had a 10-day course of amoxicillin/clavulanic acid at an appropriate dose, and an additional 4-day underdosed course which we assume it had been a dosing prescribing error. Due to financial restrictions the initial urine sample that was submitted for culture was not collected aseptically and therefore the validity of its growth in this case still remains unclear. Interestingly this ‘coliform’ growth was susceptible to marbofloxacin but not to amoxicillin/clavulanic acid. However, the 10^3^ CFU/ml was not considered significant, bearing in mind the sample was a free void sample, although it may have been affected by the concurrent use of antibiotic. It is therefore likely that this patient had two separate infections, bacterial endocarditis and urinary tract infection, from two different aetiological agents as she improved significantly following the combined antibiotherapy. A new urine culture from a cystocentesis sample obtained 10 days later, yielded no growth.

Despite the grave prognosis canine patients with aortic bacterial endocarditis carry, the patient in this case had a positive outcome and was still alive 25 months following initial diagnosis. The initial diastolic murmur was no longer audible; instead, a left-sided systolic grade IV/VI heart murmur remained present at the base of the heart. This murmur was thought to be associated with  mitral valve regurgitation, as a sequela of the left ventricular changes and/or aortic valve structural alteration. To the best of our knowledge, long term recovery from infective aortic valve endocarditis in dogs has never been reported before.

We assume that the left ventricular dilation was due to the continued aortic regurgitation. Therefore there are concerns that further ventricular dilatation will occur and may ultimately lead to heart failure. In humans *Actinomyces neuii* endocarditis is associated with a more favourable prognosis and long-term antibiotics (sometimes up to a year) are often required [12]. This case suggests that the same may be true in dogs affected with endocarditis where *Actinomyces neuii* is isolated.

## Data Availability

Available upon request, currently in the patient’s private file.

## Abbreviations

FS: Fractional shortening
PO: Orally
LVDd: Left ventricular diastolic diameter
LVDs: Left ventricular systolic diameter
